# MLST-based genetic relatedness of *Campylobacter jejuni* isolated from chickens and humans in Poland

**DOI:** 10.1371/journal.pone.0226238

**Published:** 2020-01-24

**Authors:** Kinga Wieczorek, Tomasz Wołkowicz, Jacek Osek

**Affiliations:** 1 Department of Hygiene of Food of Animal Origin, National Veterinary Research Institute, Pulawy, Poland; 2 Department of Bacteriology and Biocontamination Control, National Institute of Public Health—National Institute of Hygiene, Warsaw, Poland; Massey University, NEW ZEALAND

## Abstract

*Campylobacter jejuni* infection is one of the most frequently reported foodborne bacterial diseases worldwide. The main transmission route of these microorganisms to humans is consumption of contaminated food, especially of chicken origin. The aim of this study was to analyze the genetic relatedness of *C*. *jejuni* from chicken sources (feces, carcasses, and meat) and from humans with diarrhea as well as to subtype the isolates to gain better insight into their population structure present in Poland. *C*. *jejuni* were genotyped using multilocus sequence typing (MLST) and sequence types (STs) were assigned in the MLST database. Among 602 isolates tested, a total of 121 different STs, including 70 (57.9%) unique to the isolates' origin, and 32 STs that were not present in the MLST database were identified. The most prevalent STs were ST464 and ST257, with 58 (9.6%) and 52 (8.6%) *C*. *jejuni* isolates, respectively. Isolates with some STs (464, 6411, 257, 50) were shown to be common in chickens, whereas others (e.g. ST21 and ST572) were more often identified among human *C*. *jejuni*. It was shown that of 47 human sequence types, 26 STs (106 isolates), 23 STs (102 isolates), and 29 STs (100 isolates) were also identified in chicken feces, meat, and carcasses, respectively. These results, together with the high and similar proportional similarity indexes (PSI) calculated for *C*. *jejuni* isolated from patients and chickens, may suggest that human campylobacteriosis was associated with contaminated chicken meat or meat products or other kinds of food cross-contaminated with campylobacters of chicken origin. The frequency of various sequence types identified in the present study generally reflects of the prevalence of STs in other countries which may suggest that *C*. *jejuni* with some STs have a global distribution, while other genotypes may be more restricted to certain countries.

## Introduction

*Campylobacter jejuni* is one of the most frequently reported causes of foodborne bacterial enteric diseases worldwide, with a total of 246,307 laboratory confirmed infections (notification rate of 64.8 cases per 100,000 population) in the European Union in 2017 [1]. At the same time, only 874 campylobacteriosis cases (rate 2.3) were noted in Poland; however, the number of persons suffering from *C*. *jejuni* infection has increased during the last 10 years (270 cases in 2008), which may be partially due to increased chicken meat consumption, i.e. from 24.1 kg per capita in 2008 to 30.5 kg per capita in 2018 [https://stat.gov.pl]. Another reason of a higher number of *Campylobacter* infection cases identified during last years may be more laboratory analyses performed in patients with diarrhea. Campylobacteriosis in Poland is the disease subjected to registration under the Act of 5.12.2008 on the prevention and control of infections and infectious diseases in humans (https://www.sejm.gov.pl). However, a relatively low number of cases with a *Campylobacter* infection in Poland in comparison to neighboring countries suggests a substantial underestimation of cases, e.g. due to still inadequate diagnosis of *Campylobacter* in patients with diarrhea.

*Campylobacter* are widespread in the environment and they are a part of the natural microflora of birds and other animals. The main transmission route of these microorganisms to humans is by consumption and handling of contaminated food, especially of poultry origin [2–4]. Chicken carcasses are being frequently contaminated in the abattoirs, especially by direct contact with intestinal content of slaughtered chickens which may contain up to 10^8^ of *Campylobacter* cells per gram [2, 5]. During processing, rupturing of the viscera is common due to carcass size variation and the fixed size of evisceration machinery [6]. It has been shown that the main critical points for contamination of carcasses were plucking, evisceration, washing and air or water immersion chilling [6].

The wide distribution and high molecular diversity among *C*. *jejuni* make it difficult to compare and identify the source of infection and transmission routes [4]. There are various genotyping methods used for molecular differentiation of *Campylobacter* isolates. Among them, multilocus sequence typing (MLST) has been the most widely applied due to its high discriminatory power and reproducibility [7, 8]. MLST exploits the genetic variation present in seven housekeeping *C*. *jejuni* loci to determine the molecular relationships among isolates. This approach has been successfully used for investigation of bacteria with a weak clonal population structure and for identification of *C*. *jejuni* reservoirs and determination of transmission routes for human infection [7–9]. Furthermore, the MLST analysis can easily be applied to compare the results obtained in different studies and a *Campylobacter* open database is available at PubMLST (http://pubmlst.org/campylobacter). It has been shown that MLST typing is complementary to pulsed-field gel electrophoresis (PFGE) and whole genome sequencing methods [10].

In the present investigation, the molecular typing of a collection of *C*. *jejuni* isolates of chicken origin and from humans with diarrhea using MLST-sequencing was performed. The objectives of the study were: (i) to analyze the genetic relatedness of *C*. *jejuni* isolates obtained from chicken sources and from patients suffering from campylobacteriosis and (ii) to subtype the isolates to gain better insight into the population structure of *C*. *jejuni* recovered in Poland and to compare it with molecular variants identified in other countries. To our knowledge, this study provides information on MLST types and phylogenic relationship among *C*. *jejuni* isolated from chicken sources and humans in Poland for the first time, allowing a better understanding of subtype diversity as well as a possible source and route of *Campylobacter* human infection in this country.

## Materials and methods

### Ethics statement

The authors declare that the study did not need any recommendation or approval of ethics committee nor written consent from humans from whom *C*. *jejuni* were isolated. The isolates from the unidentifiable patients were provided for research purposes and were recovered from the stool specimens by the field microbiological laboratories during routine diagnostics. The *Campylobacter* isolates were then sent to the National Institute of Public Health—National Institute of Hygiene in Warsaw, Poland for bacterial species identification. All human isolates used in the study were anonymous, i.e. did not contain any information enabling identification of persons from whom they were recovered.

No chickens were sacrificed for the purpose of the present study. All samples were collected by well-trained official veterinarians in poultry abattoirs where chickens were slaughtered for commercial purposes. The intact ceca, swabs from the neck skin and surface under the wings of the chicken carcasses were subsequently used only for this research study.

### *C*. *jejuni* isolates

A total of 602 *C*. *jejuni* isolates collected between 2010 and 2016 in Poland were used in the study (S1 Table). The isolates from chicken feces (n = 151) were obtained using the procedure as described earlier and then confirmed with PCR [11]. Briefly, intact ceca from 10 birds were taken in slaughterhouses after evisceration and transported to the laboratory within 24 h at 2–8°C. Then, the content was pooled and one loop-full (10 μl) of the material was streaked directly on Karmali agar (*Campylobacter* Agar Base + *Campylobacter* Selective Supplement; Oxoid, Basingstoke, UK) and *Campylobacter* blood-free agar (Oxoid) with CCDA selective supplement (Oxoid) and incubated at 41.5 ± 1°C for at least 48 h in a microaerobic atmosphere generated with CampyGen kit (Oxoid). The cecal isolates were collected in all 16 voivodeships (administrative districts) of Poland.

The swab samples from broiler carcasses (n = 150, obtained from all 16 voivodeships) were collected at abattoirs directly after immersion chilling (0 to 4°C) but before further processing. The neck skin and the skin surface under the wings of broiler carcasses were wiped 10 times with sterile swabs which were then immediately transported to the laboratory in Amies transport medium with charcoal (Medlab, Raszyn, Poland). *C*. *jejuni* bacteria were isolated and PCR confirmed as described [11, 12]. Briefly, the swabs were placed in Bolton enrichment broth with vancomycin, cefoperazone, trimethoprim, and amphotericin B and incubated as above. The cultures were then plated onto Karmali agar and *Campylobacter* blood-free agar with CCDA selective supplement and incubated at the same conditions. From each sample one presumptive *Campylobacter* isolate was confirmed by PCR as described previously [11].

The *Campylobacter* isolates from chicken meat purchased in local retail shops (n = 150, collected in one voivodeship) were recovered using the ISO 10272–1 standard and *C*. *jejuni* isolates were confirmed with PCR as described for the broiler carcasses [11].

A total of 151 isolates from patients with diarrhea (isolated in 5 voivodeships) were obtained using standard culturing techniques. Rectal swabs were directly streaked onto mCCDA agar (Oxoid) and incubated at 41.5°C ± 1°C for 48 h ± 2 h under microaerobic conditions and *C*. *jejuni* was identified with PCR as described previously [13].

All isolates were stored at -80°C until further analyses. The details of the isolation and characteristics of all *C*. *jejuni* used in the present study have been described previously [14].

### Multilocus sequence typing (MLST)

MLST was carried out as described by Colles and Maiden [7] and Dingle et al. [8]. Amplifications of the seven housekeeping genes that are included in the analysis scheme (*asp*A, *gln*A, *glu*A, *gly*A, *pgm*, *tkt*, *unc*A) were separately performed in a final volume of 25 μl PCR reaction as described previously [15]. The primers used for the amplification and sequencing of the genes and the PCR protocols were available at the *Campylobacter* MLST website (http://pubmlst.org/campylobacter). The obtained sequences were imported, checked for quality and analyzed to obtain the allele identifiers and sequence types (STs) together with clonal complex (CC) information using the BioNumerics v. 7.6 software (Applied Maths, Sint-Martens-Latem, Belgium) as described previously [15]. New alleles and STs were submitted to the PubMLST database.

### Statistical analyses

The relatedness between human and chicken *C*. *jejuni* isolates was assessed using the Simpson's index of diversity (D) whereas the proportional similarity index (PSI) was applied to compare sequence types distribution among *C*. *jejuni* isolates from various sources [16, 17]. The frequency distributions of the different sources were estimated by calculating their similarity using the following equation: PSI = 1–0.5Σ_i_ │p_i_—q_i_│ = Σ_i_ min (p_i_, q_i_), where p_i_ and q_i_ are the proportion of isolates from group p and q, respectively, belonging to type i. PSI ranges from zero to one, where one indicates that two groups are identical and zero means they share no types. 95% confidence intervals (CI) were computed using bias-corrected and accelerated non-parametric bootstrap. Calculations were performed using R, ver. 3.1.3 and @RISK for Excel, ver. 6.0.1 (Palisade Co., Ithaca, N.Y.). An index greater than 0.90 is considered desirable if the typing results are to be interpreted with confidence [15]. Minimum spanning tree (MST) was generated using BioNumerics based on the MLST allele number and the predefined template for categorical data.

## Results

### MLST typing

Samples for the isolation of *C*. *jejuni* (chicken feces, carcasses or meat; human) were collected from 2010 to 2016; however, the sampling and the number of isolates obtained varied from year to year (range 47 to 128 *Campylobacter* isolates per year; Table 1). During the first year of the study only isolates from chicken meat were collected, whereas in 2011 *C*. *jejuni* of meat and human origins were obtained. Most samples were isolated in 2016, although in that year only 2 *C*. *jejuni* were collected from patients with campylobacteriosis. Detailed information on the geographical origin of *C*. *jejuni* isolates tested are shown in S1 Table.

**Table 1:** Number of *C*. *jejuni* isolates by source and year of sample collection.

Source of isolates		Year of collection and no. of isolates						
		2010	2011	2012	2013	2014	2015	2016
Chicken:	feces	0	0	0	0	77	0	74
	carcasses	0	0	30	27	30	30	33
	meat	49	19	33	0	0	30	19
Humans		0	28	38	32	2	49	2
Total		49	47	101	59	109	109	128

Analysis of STs distribution among *C*. *jejuni* in relation to chicken and human sources revealed that ST464 was the most common sequence type overall (total 58 out of 602 isolates; 9.6%). ST464 was mainly identified in 2012 and 2015 (S1 Table). Similar number of isolates were of ST257 (52 out of 602; 8.6%). These *C*. *jejuni* were also obtained both from chicken sources (29 out of 451; 6.4% isolates) and humans (23 out of 151; 15.2% isolates), mainly in years 2012–2013 (S1 Table). *C*. *jejuni* with the remaining sequence types were more often recovered from chicken samples than from patients (Table 2).

**Table 2:** Prevalence of MLST sequence types in *C*. *jejuni* from chickens and humans.

Source of isolates		MLST sequence types and number of isolates:														
		464	257	50	6461	6411	353	21	5397	572	2036	122	137	824	Other (No. of different STs)^a^	Total no. of isolates
Chicken	feces	17	8	4	9	16	6	1	13	2	2	4	0	3	66 (47)	151
	carcasses	12	10	14	9	12	6	6	1	3	1	4	1	5	66 (42)	150
	meat	5	11	9	10	2	4	3	2	4	7	4	12	1	76 (43)	150
Humans		24	23	11	7	3	5	9	1	6	5	2	0	3	52 (35)	151
Total no. of isolates		58	52	38	35	33	21	19	17	15	15	14	13	12	260 (108)	602

^a^ Other includes 108 different STs, counting from 10 to one isolate; among them are 39 isolates with 30 novel MLST sequence types.

Among 151 human *C*. *jejuni*, collected in 5 geographical districts (voivodeships), 47 sequence types were identified. The most prevalent sequence type was ST464 (24 isolates;15.9%) (S1 Table). Thirteen of 47 STs (27.7%) were unique to the human *C*. *jejuni*, whereas the remaining 34 MLST sequences (138 isolates) were also identified in chicken (Table 3). One human isolate had a new sequence type (assigned as ST8644) which had not been present in the MLST database. Furthermore, a total of 17 MLST clonal complexes (CCs) were also identified. The most prevalent one was CC257 (28 isolates; 18.5%) (Table 3).

**Table 3:** Distribution of *C*. *jejuni* MLST molecular subtypes according to the isolates' origin.

Molecular subtypes	*C*. *jejuni* origin (no. of isolates):				
	Humans (n = 151)	Chicken:			Total (n = 602)
		Feces (n = 151)	Carcasses (n = 150)	Meat (n = 150)	
No. of novel STs	1 (1)	8 (9)	8 (9)	15 (20)	32 (39)
No. of STs unique to the origin	13 (16)	19 (21)	17 (21)	21 (31)	70 (89)
No. of STs common to other origins	18 (116)	18 (94)	18 (94)	18 (71)	18 (375)
No. of CCs	17	20	19	14	22
Most prevalent CC	CC257 (28)	CC353 (26)	CC21 (24)	CC353 (34)	CC353 (107)

*C*. *jejuni* recovered from chicken feces (n = 151) in all 16 Polish administrative districts (S1 Table) were classified into 59 MLST STs (Table 2). A total of 19 STs unique to the origin (comprising of 21; 13.9% isolates) were detected whereas the remaining 40 sequence types with 130 isolates were found among other strain sources tested. Eight novel STs among *C*. *jejuni* of feces origin were identified and deposited in the MLST database. MLST analysis also revealed that isolates of chicken feces origin belonged to 20 clonal complexes (CCs), with CC353 as the most common (26; 17.2% of isolates) (Table 3).

Isolates originating from chicken carcasses (n = 150), collected in all 16 voivodeships (S1 Table), were classified to 55 STs, with ST50 as the predominant one (14; 9.3% isolates). *C*. *jejuni* with this sequence type were also often identified among human (11; 7.3% isolates) and chicken meat (9; 6.0% isolates) isolates, respectively. On the other hand, 8 new STs (covering 9 isolates) unique to the chicken carcasses were detected (Table 3). Additionally, 19 clonal complexes were identified among *C*. *jejuni* isolates from chicken carcasses. The most prevalent one was CC21 (24; 16.0% isolates) (Table 3).

*C*. *jejuni* isolated from chicken meat (n = 150; all collected in one administrative district–lubelskie voivodeship; S1 Table) belonged to 56 STs. ST137 was the most frequently detected sequence type in chicken meat isolates (12; 8.0% isolates). Interestingly, only one isolate (from broiler carcass) with such sequence type was identified among the remaining 452 isolates tested from chicken carcasses, chicken feces, and humans. Fifteen new STs covering 20 isolates were detected among *C*. *jejuni* of chicken meat origin (Table 3). MLST analysis also showed that all isolates of this origin were classified into 14 clonal complexes, with the predominant CC353 (34; 22.7% isolates) (Table 3).

It was found that of 47 human STs, 26 sequence types (106 isolates) were also identified among chicken feces, 23 STs (102 isolates) in chicken carcasses, and 29 STs (100 isolates) in chicken meat, respectively (S1 Table). The same STs of the isolates recovered from feces were detected among isolates of human (26 STs; 125 isolates), chicken carcasses (31 STs; 117 isolates), and chicken meat (26 STs; 87 isolates) origins. Analysis of the sequence types of *C*. *jejuni* of carcass origin revealed that isolates with common STs were identified in the bacteria recovered from humans (23 STs; 123 isolates), chicken feces (31 STs; 116 isolates), and chicken meat (24 STs; 93 isolates). Similar comparison of the strains of meat origin revealed that the bacteria with the same sequence types were also detected among *C*. *jejuni* of human (29 STs; 129 isolates), chicken feces (26 STs; 105 isolates), and chicken carcasses (24 STs; 105 isolates) origins, respectively (S1 Table).

### Population structure of chicken and human *C*. *jejuni* isolates

**Fig 1:**
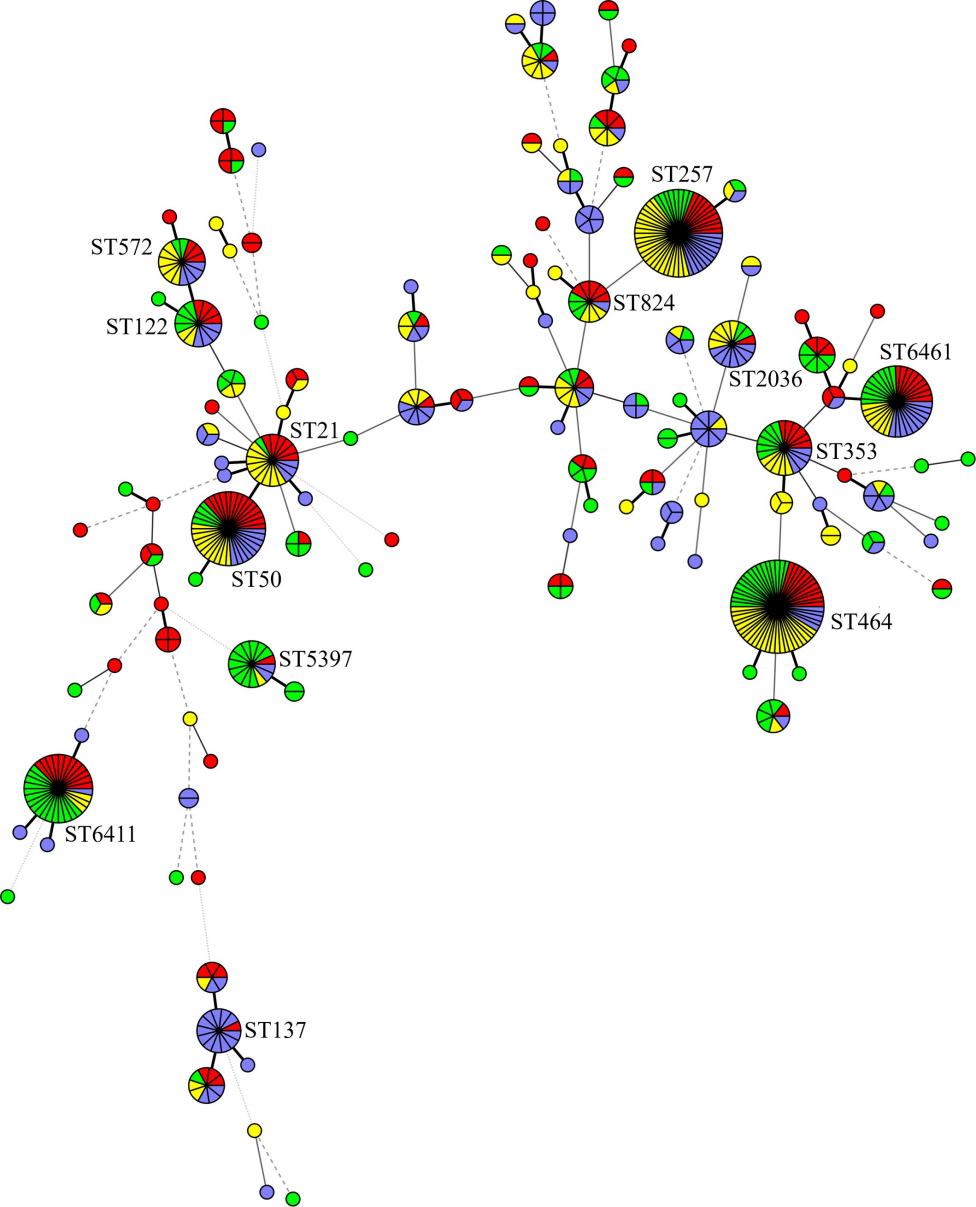
Minimum spanning tree established on the 117 identified MLST sequence types (STs) of 602 *C*. *jejuni* isolates obtained from chicken feces, carcasses, meat, and humans. Each circle and number by the circle represents one ST. The size of the circle is positively correlated to the isolate numbers with the same ST and different colours indicate isolate source (green—chicken feces, blue—chicken carcasses, red—chicken meat, yellow—humans). The thickness of the circle connecting lines is proportional to the similarities between STs.

Overall, a total of 121 MLST sequence types were identified, including 70 (57.9%) unique to the isolates' origin (Table 3). Among them, 30 STs detected in 39 isolates were not present in the MLST database. The most common STs found in all 602 isolates tested were ST464 and ST257, which included 58 (9.6%) and 52 (8.6%) *C*. *jejuni* isolates, respectively. The remaining numbers of isolates belonging to particular STs are shown in Table 2. The minimum spanning tree, generated from the MLST typing data based on the STs, showing the phylogenetic relationship of all 602 *C*. *jejuni* isolates, is presented in Fig 1.

### Discriminatory power of MLST

Overall, the MLST typing method was highly discriminatory for all *C*. *jejuni* used in the study since the Simpson's diversity index (D) achieved value 0.966, indicating considerable diversity in the bacterial population tested (Table 4). Taking into account the number of STs, the method was more discriminatory in case of *C*. *jejuni* isolated from chicken samples (D from 0.959 for fecal strains to 0.971 for meat isolates) than for human isolates (D 0.937), i.e. isolates collected from chickens showed a higher genetic diversity than the bacteria from patients, although the confidence intervals indicated that these differences were not statistically significant.

**Table 4:** Molecular diversity of *C*. *jejuni* isolates based on Simpson's diversity (D) and proportional similarity indexes (PSI).

Source od isolates		No. of isolates (sequence types)	PSI (95% CI)^a^					D (95% CI)
			Humans	Chicken:				
				feces	carcasses	meat	all sources	
Humans		151 (47)	1	-	-	-	-	0.937 (0.917–0.957)
Chicken:	feces	151 (59)	0.843 (0.805–0.874)	1	-	-	-	0.961 (0.948–0.974)
	carcasses	150 (55)	0.841 (0.806–0.872)	0.834 (0.799–0.865)	1	-	-	0.965 (0.955–0.976)
	meat	150 (56)	0.843 (0.805–0.875)	0.826 (0.787–0.861)	0.827 (0.793–0.860)	1	-	0.971 (0.963–0.979)
	all sources	451 (104)	0.843 (0.814–0.870)	NA^b^	NA	NA	1	0.971 (0.966–0.976)

^a^ CI, confidence intervals with 95% confidence level. 1 = maximal similarity; 0 = maximal difference

^b^ NA, not applicable

The PSIs and their 95% CIs were calculated to assess the similarity of ST distributions between different *C*. *jejuni* sources, i.e. humans and chicken sources, i.e. feces, carcasses, and meat (Table 4). The MLST sequence types identified in the chicken samples were highly similar (PSIs above 0.8) and the similarity of the poultry and human isolates was also at the comparable levels.

## Discussion

In the present study an extensive genotypic investigation of a large group of *C*. *jejuni* isolates of chicken and human origins was performed. Such study has never been done in Poland before and revealed, based on the MLST analysis, a high molecular diversity of *C*. *jejuni* isolated from chicken sources and from humans with diarrhea. Isolates with some STs (e.g. 464, 6411, 257, 6411, 50) were commonly identified in chickens, whereas the other sequence types were more often detected among human isolates (e.g. ST21, ST50, ST572). However, the isolates classified to all mentioned STs were found within *C*. *jejuni* recovered from all sources tested. The similarities in the distribution of the same STs between chicken and human isolates suggest that at least some cases of campylobacteriosis were associated with the consumption of contaminated chicken meat or meat products or other kinds of food cross-contaminated with campylobacters. Another possibility may be direct contact of persons with chickens carrying *C*. *jejuni*. However, the human *Campylobacter*-positive samples tested were originated from 5 out of 16 voivodeships (administrative districts) in Poland whereas chicken meat samples were purchased only in one voivodeship. Furthermore, *Campylobacter*-contaminated meat was not tested during 2013–2014, when a total of 34 positive samples from humans were identified. Therefore, it is difficult to draw the conclusion that the source of human infection in the voivodeships where patients were tested was chicken meat containing *C*. *jejuni*, at least in those two years, when no sample was investigated towards *Campylobacter*.

Our previous study demonstrated that *C*. *jejuni* strains with ST6411 and ST257 were predominant among chickens in Poland, representing a total of 31.5% isolates tested [15]. These sequence types were also often identified in chicken sources in the present investigation although they were not as common as before (a total of 58 out of 451 isolates; 12.7%). It was also previously found that *C*. *jejuni* with ST137 were mainly identified among human isolates whereas only one such strain was detected in campylobacters of chicken origin [15]. Some authors suggest that certain *C*. *jejuni* molecular variants are more sensitive to stress conditions, e.g. present along poultry food production and are not able to survive in the environment and during food processing, although they may infect humans from other sources than poultry [18, 19]. Furthermore, isolates with certain STs are often identified in chickens, which is recognized as the main source of human infection, but rarely or not at all detected in infected patients, e.g. ST6411 or ST5397 in the present study.

Previous studies in Europe demonstrated that ST45, ST50, ST573, and ST2274 were often identified in *C*. *jejuni* from chickens [20–23]. Some of these sequence types (i.e. ST45, ST50) were also detected in the present investigation. A comprehensive analysis on MLST genotypes of *C*. *jejuni* isolated from broiler products in Lithuania, a country neighboring Poland, demonstrated that ST50 was also one of the commonly identified variant as found in the current study [24]. Investigations performed in other geographical regions indicated that *C*. *jejuni* of chicken origin had distinct as well as the same MLST variants than those identified in Europe. For example, ST5, ST4526, ST5742, ST6422, ST7669 were detected in Asia as well as in Europe [25, 26]. However, these STs were not detected in our study. *C*. *jejuni* ST50, ST257, and ST464, which are typically of chicken origin, were found in distant geographical regions such as New Zealand [27], but are also common in Europe [http://pubmlst.org/campylobacter], including Poland, as demonstrated in our study.

The sequence types ST50 and ST257 are widespread in chicken and in chicken meat and in samples from other poultry as well as in the isolates from other sources in many countries (http://pubmlst.org/campylobacter). In this database (access 08.05.2019) there is information of a total of 17,429 isolates of poultry origin and among them are 748 (4.3%) with ST50 and 541 (3.1%) with ST257 but only 114 (0.6%) with ST464 which was the most commonly identified in the present investigation. At the same time, only 265 of chicken *C*. *jejuni* strains from Poland were found in PubMLST, which were mainly classified to ST6411 (28; 10.6%) and ST257 (23; 8.7%). As reported by other authors, several MLST *C*. *jejuni* sequence types may represent local clones restricted to one country or source of isolates [28]. Furthermore, it has been suggested that certain *Campylobacter* genotypes might have been able to circulate at a geographical area and respond better than others to stress conditions during food processing. On the other hand, other *C*. *jejuni* MLST variants were never recovered from chicken carcasses despite being present in the broilers [29, 30]. However, the reasons for the predominance of particular *C*. *jejuni* genotypes are clearly not known but may be explained by factors such as climate, geography or over-representation of certain MLST types in the environment [18].

There is a limited number of studies on MLST sequence types of *C*. *jejuni* from humans, but a very diverse populations of such isolates, similarly like chicken *C*. *jejuni*, were identified. Studies performed in Europe showed that there are predominant STs circulating in patients suffering from campylobacteriosis, i.e. ST21, ST22, ST45, ST48, ST53, ST257, ST267 [22, 23, 31–35]. In the mentioned studies of Ramonaite et al. [24] performed in Lithuania *C*. *jejuni* of human clinical isolates were mainly classified to ST5, ST50, and ST227 (a total of 42 out of 117; 35.9% isolates). In the current investigation, *C*. *jejuni* with the sequence type 50 was also identified, although in a smaller percentage than in Lithuania, i.e. 7.3% as compared to 9.4%, respectively. *C*. *jejuni* classified to some of these molecular variants were also identified among humans in Poland during the present study (e.g. ST21, ST50, ST257; total 20 of 151, 13.2% isolates), which may suggest that isolates with these sequence types are more virulent that the others.

Outside Europe, several other *C*. *jejuni* MLST variants were detected (e.g. ST48, ST478, ST528, ST3219, ST4526) which had not or very seldom (i.e. ST45; one isolate) been identified in Poland during the present investigation [23, 36, 37].

In the MLST database (access 08.05.2019) there is information of a total of 38,924 isolates from humans, including only 10 *C*. *jejuni* from Poland (http://pubmlst.org/campylobacter). Many strains were classified into ST50 (2,082 isolates; 5.3%) and ST257 (2,021 isolates; 5.2%). *C*. *jejuni* with these sequence types were also often identified in the present study, i.e. among 7.3% and 15.2% of isolates from patients with diarrhea, respectively. However, it is important to note that the isolate population in the database does not reflect the true prevalence and source distribution due to the sampling and reporting biases.

Several studies comparing isolates from patients suffering from campylobacteriosis and from various food animals found a high overlap of human with chicken isolates [9, 23, 25, 27, 33, 35, 38–42]. In the present study, *C*. *jejuni* with sequence types ST464, ST257, ST50, and to a lesser extent, other STs were shared among chicken and human isolates. As mentioned above, strains with these STs are relatively widely distributed across many countries not only in Europe but also in Australia and Asia [9, 23, 26]. Other STs (i.e. ST6461, ST6411, ST5397) were more common in chickens than in patients with diarrhea. Such clonal population structures of the isolates may be due to source and geographic adaptation, host immune selection, or barriers to genetic exchange [43]. According to the PubMLST database, strains with ST464, ST257, and ST50 were identified among both chicken and human sources (http://pubmlst.org/campylobacter). Furthermore, in this database 6.6% of human and 1.8% of chicken *C*. *jejuni* belonged to ST21 which was detected in 6.0% and 2.2% isolates in the present study, respectively.

## Conclusions

The results of the present study showed that *C*. *jejuni* of chicken origin and isolated from humans represent a high level of genetic diversity as tested by MLST, although certain sequence types were predominant in Poland, either in chicken (ST464, ST257, ST50, ST6461, ST6411) or in human populations (ST464, ST257, ST50, ST21). Isolates with some of these sequence variants were also identified in other European countries (e.g. ST50, ST257) which may suggest that such strains are circulating in chickens and humans suffering with campylobacteriosis regardless of the place of origin. The high similarity values observed among *Campylobacter* isolates from all sources (PSIs above 0.8) suggests a strong link between chicken isolates and human campylobacteriosis cases tested in the study, although the source of human infections has not been described. The identified overlap of *C*. *jejuni* STs recovered from patients and chickens highlights the importance of this source for human campylobacteriosis in Poland as in many other countries. The genetic structure of the isolates identified in the present study generally reflects of the distribution of STs in other countries. However, a total of 32 new MLST sequence types were found and included into the public database becoming available for other researchers and epidemiologists. Overall, our findings provide new insights into the distribution of MLST *C*. *jejuni* variants among different chicken sources and humans in Poland. The obtained results may suggest that *C*. *jejuni* with some STs have a global distribution, while other genotypes may be more restricted geographically. Such examination of isolates from different geographical locations provides further insight into the epidemiology and population structure of *Campylobacter*. However, more such investigations are needed in a future.

## Supporting information

S1 Table Characteristics of *C*. *jejuni* isolates used in the study. All information related to isolation dates, administrative regions, slaughterhouses, MLST STs, MLST CCs are given.
(XLSX)
